# Kaposi Sarcoma–Associated Herpesvirus Infection and Complications Among Solid Organ Transplant Recipients — United States, January 2021–September 2025

**DOI:** 10.15585/mmwr.mm7508a1

**Published:** 2026-03-05

**Authors:** Ian Kracalik, Pallavi Annambhotla, David W. McCormick, Andrew I. Geller, Kelsey McDavid, Isabel Griffin, Raymond Lynch, Brianna Doby, Yoichiro Natori, Sofya Tokman, Christine M. Durand, Camille N. Kotton, Emily Blumberg, Ricardo M. La Hoz, Lauri A. Hicks, Stephanie M. Pouch, Sridhar V. Basavaraju, Donor-Derived KSHV Investigation Group, Donor-Derived KSHV Investigation Group, Adam P. Bregman, Costi D. Sifri, Megan Del Vecchio, Amir Emtiazjoo, Ghady Haidar, Kenneth T. Hughes, Nicholas Marschalk, Rachel A. Miller, Swati Rao, Mary Saputo, Benjamin Keebler, Darryl Nethercot, Matthew Niles, Patricia Carroll, Kerri Jones

**Affiliations:** ^1^Division of Healthcare Quality Promotion, National Center for Emerging and Zoonotic Infectious Diseases, CDC; ^2^Division of Transplantation, Health Services Bureau, Health Resources and Services Administration, Rockville, Maryland; ^3^University of Miami School of Medicine, Miami, Florida; ^4^Creighton School of Medicine, Norton Thoracic Institute, St. Joseph’s Hospital and Medical Center, Phoenix, Arizona; ^5^Johns Hopkins University School of Medicine, Baltimore, Maryland; ^6^Massachusetts General Hospital, Boston, Massachusetts; ^7^University of Pennsylvania Perelman School of Medicine, Philadelphia, Pennsylvania; ^8^University of Texas Southwestern Medical Center, Dallas, Texas; ^9^Emory University School of Medicine, Atlanta, Georgia.; University of Wisconsin-Madison School of Medicine and Public Health; University of Virginia Transplant Center; University of California, San Diego; University of Florida Transplant Center; University of Pittsburgh; University of Texas Health San Antonio; The Ohio State University Medical Center; Duke University; University of Virginia; New York University; Southwest Transplant Alliance; Lifesharing; Network for Hope; LifeLink of Florida; New England Donor Services.

**Summary**

**What is already known about this topic?**

Kaposi sarcoma–associated herpesvirus (KSHV) is the cause of Kaposi sarcoma and certain lymphoproliferative disorders. In solid organ transplant recipients, KSHV-related complications can result from reactivation of latent infection, new posttransplant infection, or transmission of virus from the donated organ.

**What is added by this report?**

During January 2021–September 2025, 46 cases of suspected donor-derived KSHV-related complications were reported among transplant recipients, compared with nine during 2016–2020. Most donors and recipients were HIV-negative, and two thirds of donors had a history of nonmedical inhalation or injection drug use.

**What are the implications for public health practice?**

Maintaining a high level of suspicion for KSHV infection by clinicians caring for organ transplant recipients could facilitate prompt diagnosis and reporting. Development of donor screening assays could help guide clinical management to mitigate recipient KSHV-related complications.

## Abstract

Kaposi sarcoma–associated herpesvirus (KSHV) infection is the cause of Kaposi sarcoma (KS), certain lymphoproliferative disorders, and the inflammatory condition Kaposi sarcoma–associated herpesvirus inflammatory cytokine syndrome (KICS). In solid organ transplant recipients, KSHV-related complications can result from reactivation of latent infection, new posttransplant infection, or transmission of virus from the transplanted organ. However, testing of donors and recipients is not routinely performed. During January 2021–September 2025, after transplantation of 185 organs into 153 recipients, 46 deceased donors were identified whose transplanted organs were suspected of having transmitted KSHV, approximately five times the number of such donors (nine) reported during 2016–2020. As of February 2026, a posttransplantation KSHV infection has been identified among 74 (48%) of these 153 transplant recipients. Among the 74 recipients with KSHV infection, 45 (61%) developed KS; 10 (14%) of these recipients with KS also developed a lymphoproliferative disorder (multicentric Castleman disease [eight], posttransplant lymphoproliferative disorder [one], and primary effusion lymphoma [one]) and six (8%) developed KICS; four (5%) recipients developed a lymphoproliferative disorder alone (primary effusion lymphoma [one] and posttransplant lymphoproliferative disorder [three]); and one (1%) developed KICS alone. To date, 25 (16%) of the 153 transplant recipients have died. Most donors and recipients were HIV-negative, and nonmedical drug use was common among donors. Clinicians should maintain a high index of suspicion for KSHV in transplant recipients, particularly when donors have risk factors including nonmedical drug use, or when another recipient from the same donor is found to be infected. Development and implementation of effective testing strategies and timely reporting could guide clinical management, reduce risk for KSHV-related complications, and improve transplant safety.

## Introduction

Infection with Kaposi sarcoma–associated herpesvirus (KSHV), also known as human herpesvirus 8, is the cause of Kaposi sarcoma (KS), certain lymphoproliferative disorders (including multicentric Castleman disease and primary effusion lymphoma), and Kaposi sarcoma–associated herpesvirus inflammatory cytokine syndrome (KICS), a recently described inflammatory condition resembling severe sepsis that affects persons infected with KSHV (*1*,*2*). In solid organ transplant recipients, posttransplantation KSHV-related complications can result from 1) reactivation of latent infection, 2) new posttransplantation infection, or 3) transmission of KSHV from the transplanted organ (*3*). Because transplant recipients receive immunosuppressive medication to prevent graft rejection, infection in these persons can be severe and result in death. However, although testing of deceased donors is required for certain infectious diseases, testing of donors and recipients for KSHV is not routinely performed, given the limited availability of commercially available tests and the current absence of consensus screening guidelines.

In 2021, CDC reported KSHV transmission through solid organ transplantation involving six donors investigated during 2018–2020 (a subset of nine total cases referred during that time). In these clusters, four (29%) of 14 recipients who developed donor-derived infection died (*3*).

During January 2021–September 2025, transplanted organs from 46 deceased donors were suspected of having transmitted KSHV, approximately five times the nine such cases reported during 2016–2020. To date, a total of 185 organs implicated in KSHV transmission have been transplanted into 153 recipients from these 46 deceased donors. Among transplant recipients, a posttransplantation KSHV infection has been identified in 74 (48%). This report describes preliminary findings from ongoing CDC investigations of suspected solid organ donor–derived KSHV infections and associated complications in U.S. transplant recipients. Additional interventions are necessary to reduce the risk for transplant-associated KSHV complications.

## Methods

### Data Source

Transplant centers are required to report any suspected, unexpected organ donor–derived infectious disease or malignancy to the Organ Procurement Transplantation Network (OPTN).* CDC investigated all reports of suspected organ donor–derived KSHV infection based on review of medical records and laboratory testing of donor and recipient specimens that were referred to OPTN during January 2021–September 2025.

### Data Analysis

A descriptive analysis was conducted based on abstraction and review of donor and transplant recipient medical records, including data on age, sex, sexual orientation, underlying medical conditions, HIV infection status, Public Health Service risk factors (including men who have sex with men [MSM] and incarceration for ≥72 hours), and risk factors for KSHV transmission, including history of nonmedical inhalation or intravenous drug use (*4*–*6*). Donor archived serum or plasma and recipient tissue, serum, or plasma specimens were tested for KSHV by commercial laboratories, academic tertiary referral centers, or other reference laboratories using serologic, molecular, or immunohistochemical assays. This activity was reviewed by CDC, deemed not research, and was conducted consistent with applicable federal law and CDC policy.^†^

## Results

### Reported Cases of Posttransplant KSHV-Related Complications

**FIGURE 1:**
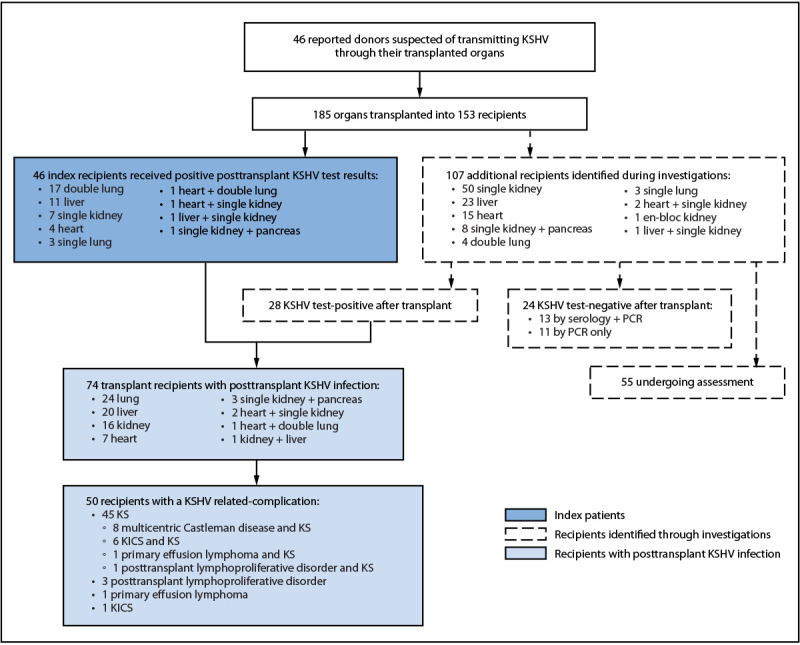
Reports of organ donor–derived Kaposi sarcoma–associated herpesvirus among transplant recipients (n = 46) and investigation of additional recipients* — United States, January 2021–September 2025 **Abbreviations:** KICS = Kaposi sarcoma–associated inflammatory cytokine syndrome; KS = Kaposi sarcoma; KSHV = Kaposi sarcoma–associated herpesvirus; PCR = polymerase chain reaction. * Retrospective KSHV testing of the 46 donors whose organs were associated with posttransplant donor organ KSHV infection identified 25 (54%) who received positive results and four (9%) who received negative results; organs from 17 (37%) donors had not been tested.

**FIGURE 2:**
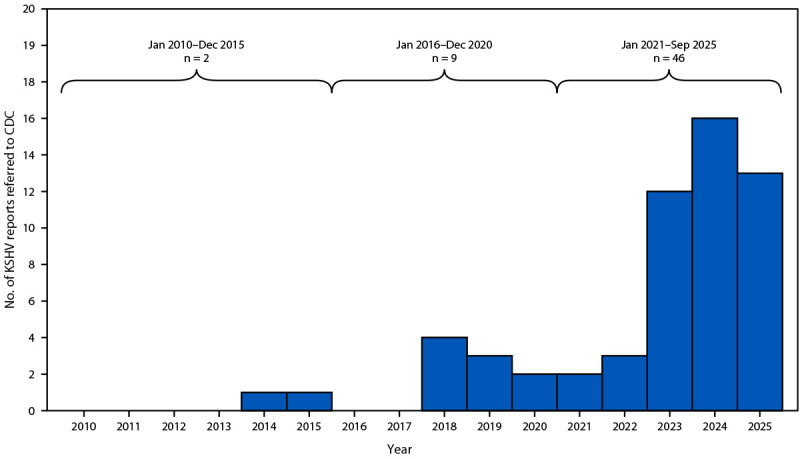
Number of reports* of suspected organ donor–derived Kaposi sarcoma–associated herpesvirus infections in transplant recipients (N = 57) — United States, January 2010–September 2025^†^ **Abbreviation:** KSHV = Kaposi sarcoma–associated herpesvirus. * Each report represents the index recipient first identified with a KSHV-related complication. ^†^ Partial year of data through September 2025.

A total of 185 solid organs implicated in KSHV transmission were recovered from 46 deceased donors and transplanted into 153 recipients (Figure 1). During January 2021–September 2025, CDC received 46 reports of transplant recipients who developed KSHV-related complications suspected to be derived from organ donors (index recipients), representing an approximately 500% increase over the nine cases reported during the previous 5-year period (2016–2020) (Figure 2). During investigation of these cases, an additional 28 organ recipients with KSHV-related complications were identified; follow-up of other recipients from these 46 donors, including clinical and KSHV infection status, is ongoing (Figure 1).

### Donor Characteristics

The median age of the 46 deceased donors was 38.5 years (IQR = 31–51 years), 67% (31) were male, 33% (15) were MSM, and 96% (44) were HIV-negative^§^ (Table). Thirty-one (67%) donors had a history of nonmedical inhalation^¶^ or injection drug use, and eight (17%) had a history of incarceration. Of the 29 donors who had testing completed after organ procurement, 25 (86%) received a positive molecular or serologic KSHV test result and four (14%) received negative test results by both assays.

**TABLE:** Characteristics of organ transplant recipients and deceased solid organ donors whose organs are suspected of having transmitted Kaposi sarcoma–associated herpesvirus — United States, January 2021–September 2025

Characteristic	No. (column %)	
	Donor n = 46	Recipient n = 153
Median age, yrs (IQR)	38.5 (31–51)	58.5 (49–65)
Male sex	31 (67)	76 (50)
Men who have sex with men	15 (33)	2 (1)
HIV-negative	44 (96)	150 (98)
History of nonmedical inhalation or injection drug use	31 (67)	NA
History of incarceration	8 (17)	NA
**Testing completed after organ procurement**	**29 (64)**	87 (57)*
Negative molecular or serologic KSHV test result	4 (14)^†^	13 (15)^†^
Positive molecular or serologic KSHV test result	25 (86)^†^	74 (85)^†,§^
Positive recipient KSHV test result by organ received, n/N (%)		
Lung	NA	24/28 (86)
Liver	NA	20/35 (57)
Heart	NA	7/23 (30)
Kidney	NA	16/72 (22)
**Recipient death**	**NA**	**25/153 (16)**
**Postinfection KSHV-related complication, n/N (%)**		
Kaposi sarcoma	NA	45/153 (29)
Multicentric Castleman disease and Kaposi sarcoma^¶^	NA	8/45 (18)
KICS and Kaposi sarcoma^¶^	NA	6/45 (13)
Posttransplant lymphoproliferative disorder and Kaposi sarcoma^¶^	NA	2/45 (4)
Posttransplant lymphoproliferative disorder	NA	4/153 (3)
KICS	NA	1/153 (1)

**Abbreviations:** KICS = Kaposi sarcoma–associated inflammatory cytokine syndrome; KSHV = Kaposi sarcoma–associated herpesvirus; NA = not applicable.

* Testing of all 153 recipients is incomplete and ongoing.

^†^ Percentages calculated from among those who had received testing as of February 2026 (i.e., 29 donors and 87 recipients).

^§^ Includes 46 (62%) index recipients and 28 (38%) recipients identified through investigation.

^¶^ Percentages calculated from among the 45 recipients who received a diagnosis of Kaposi sarcoma.

### Recipient Characteristics

The median age of the 153 transplant recipients was 58.5 years (IQR = 49–65 years), 50% (76) were male, 1% (two) were MSM, and 98% (150) were HIV-negative. Among all 153 recipients, 48% (74, including 30% [46] of index recipients and 16% [28] identified through investigation) received a positive posttransplant KSHV test result by molecular, serologic or immunohistochemical assay, and 8% (13) received negative results on at least two assays.

The highest percentage of recipients who received a positive KSHV test result posttransplant (86%) included those who received a lung from a donor whose organs were suspected of having transmitted KSHV, followed by recipients of a liver (57%), heart (30%), or kidney (22%). Among the 74 recipients with KSHV infection, 45 (61%) developed KS; 10 (14%) of these recipients with KS also developed a lymphoproliferative disorder (multicentric Castleman disease [eight], posttransplant lymphoproliferative disorder [one], and primary effusion lymphoma [one]) and six (8%) developed KICS; four (5%) recipients developed a lymphoproliferative disorder alone (primary effusion lymphoma [one] and posttransplant lymphoproliferative disorder [three]); and one (1%) developed KICS alone. To date, 25 (16%) recipients have died, although the relative contribution of KSHV to these patient deaths remains under investigation. The median interval from date of transplantation to initial clinical manifestation was 208 days (IQR = 162–332 days).

## Discussion

CDC-led investigations first identified an increase in reports of suspected organ donor–derived KSHV infection during 2018–2020 (*3*). Since then, reports of suspected organ donor–derived KSHV infections and related complications among transplant recipients have continued to increase. KSHV transmission in the United States has historically been associated with MSM or with persons with HIV (*1*,*3*); however, in this series of cases, most organ donors and recipients were HIV-negative and were not MSM. Nonmedical injection and inhalation drug use have been increasingly recognized as a risk factor for KSHV transmission (*3*,*4*,*6*). In the United States, the percentage of all deceased donors whose mechanism of death was acute drug intoxication increased from 4% in 2010 to a peak of 17% in 2023, likely reflecting the impact of the opioid epidemic. A history of substance abuse in organ donors might contribute to increased risk for KSHV transmission to recipients, although this association might be confounded by undisclosed sexual behaviors.

Limited commercial availability of KSHV assays, particularly serology, has hindered surveillance and tracking of donor-derived infections (*7*). Strategies are needed to increase testing capacity to enable routine organ donor screening and could help mitigate KSHV-related complications among transplant recipients. Clinicians caring for solid organ transplant recipients should maintain a high index of suspicion for KSHV and related complications including KICS, symptoms of which might be similar to those of culture-negative sepsis (*1*), and consider testing when 1) donors have risk factors for KSHV, 2) donor KSHV infections are identified, or 3) another transplant recipient who received an organ from the same donor has evidence of KSHV infection. When histopathological evaluation is unavailable, transplant recipient testing should include both molecular and serologic assays when possible; at a minimum, serologic testing should be performed to detect infection because 1) molecular assays might not identify recipient infections and 2) the ability to detect KSHV DNA in blood is episodic (*8*).

### Limitations

The findings in this report are subject to at least four limitations. First, only reports of suspected organ donor–derived KSHV-related complications were investigated; because investigation requires clinical suspicion that the infection was associated with the donor organ, cases might have been underreported. Second, information about deceased organ donors is reliant on next-of-kin interviews, which might not accurately capture certain behavioral characteristics. Third, not all transplant recipients were tested for KSHV using both a molecular and a serologic assay, and some recipients might decline testing, which could have lead to an underestimation of the true number of recipient infections in this report. Finally, KSHV infection in the donor does not rule out recipient reactivation or new posttransplant infection. Additional testing of recipient pretransplant specimens could help to better elucidate the role of donor-derived infection.

### Implications for Public Health Practice

Reports of donor-derived KSHV infection are relatively uncommon. Clinicians and transplant centers should promptly report suspected donor-derived KSHV infections to OPTN. In general, the benefits of transplantation outweigh the risk for infection, with donor-derived transmission occurring among fewer than 0.5% of all transplant recipients (*9*). The number of persons awaiting transplantation far exceeds the number of available organs. Organs from donors with risk factors for infectious diseases, including KSHV, may still be used safely (*10*). This public health investigation is currently ongoing with additional donor and recipient testing results pending. CDC is working with partners to develop strategies to enhance transplantation safety and reduce the impact of KSHV infection.

## Footnotes

* The OPTN Disease Transmission Advisory Committee investigates reports of unexpected transmission of infection or malignancy through solid organ transplantation. Transplant centers are required to report any suspected, unexpected organ donor–derived infectious disease or malignancy to OPTN. A subset of these cases are investigated by CDC, including those with substantial public health significance or severe outcomes, or involving multiple transplant recipients.

† 45 C.F.R. part 46.102(l)(2), 21 C.F.R. part 56; 42 U.S.C. Sect. 241(d); 5 U.S.C. Sect. 552a; 44 U.S.C. Sect. 3501 et seq.

§ The HIV Organ Policy Equity Act (HOPE Act) allows for transplantation of organs from HIV-positive donors to HIV-positive recipients.

¶ Inhalation drug use included documented use of methamphetamine, cocaine, marijuana, or alkylnitrates.
